# Interspecies control of development during mammalian gastrulation

**DOI:** 10.1042/ETLS20230083

**Published:** 2023-11-07

**Authors:** Luke Simpson, Ramiro Alberio

**Affiliations:** School of Biosciences, University of Nottingham, Sutton Bonington Campus, Loughborough LE12 5RD, U.K.

**Keywords:** cell differentiation, cell fate, embryogenesis, gastrulation, gastruloids, RNAseq

## Abstract

Gastrulation represents a pivotal phase of development and aberrations during this period can have major consequences, from minor anatomical deviations to severe congenital defects. Animal models are used to study gastrulation, however, there is considerable morphological and molecular diversity of gastrula across mammalian species. Here, we provide an overview of the latest research on interspecies developmental control across mammals. This includes single-cell atlases of several mammalian gastrula which have enabled comparisons of the temporal and molecular dynamics of differentiation. These studies highlight conserved cell differentiation regulators and both absolute and relative differences in differentiation dynamics between species. Recent advances in *in vitro* culture techniques have facilitated the derivation, maintenance and differentiation of cell lines from a range of species and the creation of multi-species models of gastrulation. Gastruloids are three-dimensional aggregates capable of self-organising and recapitulating aspects of gastrulation. Such models enable species comparisons outside the confines of the embryo. We highlight recent *in vitro* evidence that differentiation processes such as somitogenesis and neuronal maturation scale with known *in vivo* differences in developmental tempo across species. This scaling is likely due to intrinsic differences in cell biochemistry. We also highlight several studies which provide examples of cell differentiation dynamics being influenced by extrinsic factors, including culture conditions, chimeric co-culture, and xenotransplantation. These collective studies underscore the complexity of gastrulation across species, highlighting the necessity of additional datasets and studies to decipher the intricate balance between intrinsic cellular programs and extrinsic signals in shaping embryogenesis.

## Introduction

Gastrulation is a critical juncture in embryogenesis, where a seemingly simple isotropic group of cells undergo complex morphological and differentiation processes resulting in a multi-layered, spatially organised entity -the gastrula. Throughout this process factors such as cell migration, proliferation, differentiation, and apoptosis are carefully controlled to establish the three primary germ layers and fundamental body plan. Coordination between individual cells, orchestrated by complex networks of genetic and epigenetic factors, extra and intra-cellular signals, directs cell motility, specification, and behaviour. Even minor aberrations can lead to major developmental consequences, manifesting in myriad forms from minor anatomical deviations to severe congenital defects and pregnancy loss [1–3]. However, despite the integral nature of this period of development, the diversity of the gastrula across species is substantial. Indeed, this is particularly pronounced in mammals, even among closely related species there are marked differences including implantation times, embryo and extra-embryonic tissue morphology and size [4–6]. While these differences are ultimately encoded within the genome, epigenetic, transcriptional, and cell-extrinsic factors will determine any individual cell fate within an organism.

Until recently, the understanding of interspecies developmental control has been relatively limited; in mammalian embryology this has generally been due to the fact that non-rodent models have seldom been studied in detail. Consequently, most of interspecies’ differences described tend to be limited to a single mechanism and are largely qualitative. However, the advent of high-throughput sequencing technologies has revolutionised the field of developmental biology, providing an opportunity to study genetics, epigenetics, and transcription during gastrulation at an unprecedented resolution *in vivo,* that facilitates comparative analyses with less accessible embryos. This, combined with recent advances in *in vitro* culture techniques and availability of embryonic stem cells (ESCs) from multiple mammals, has enabled quantitative comparisons providing new insights into conserved and divergent regulatory events during gastrulation. This review aims to provide an overview of the current state of knowledge regarding the developmental control of gastrulation in different mammalian species, drawing on recent breakthroughs and novel research approaches.

## Gastruloids highlight conserved programs of development in mammals

Gastruloids are three-dimensional aggregates of ESCs that have the inherent capacity to self-organise into polarised structures that resemble gastrulating embryos [7,8]. Gastruloids derived from mouse ESCs (mESCs) demonstrated collective behaviours reminiscent of the posterior cells in the early mouse embryo, such as symmetry breaking, axial organisation, germ-layer specification, and axial elongation [9]. Remarkably, these behaviours can be induced relatively simply by pulsing mESC aggregates with the small molecule Wnt agonist CHIR99021 (Chi). While initially, naïve cells require a short period after aggregation to transition to a ‘primed' pluripotent state, Chi stimulation appears to be sufficient in this case to recapitulate the canonical Wnt signalling cascade which *in vivo* is initiated by Wnt3 in response to Bmp4 [10]. Comparisons with *in vivo* data suggested that after 48 h in culture, gastruloids were transcriptionally comparable to an E6.5 epiblast (early gastrula). At 144 h, gastruloids aligned to an E8.5 embryo (∼8–12 somite stage). This demonstrates that despite the complexity of the embryonic signalling environment, relatively homogeneous pluripotent cells have an intrinsic ability to self-organise and recapitulate many aspects of gastrulation when given a single signalling cue. It is important to note however, that several factors, including the formulation of the culture medium, exposure timing, and initial aggregate size, can influence the cellular composition of gastruloids experiments [7,8].

**Figure 1.**
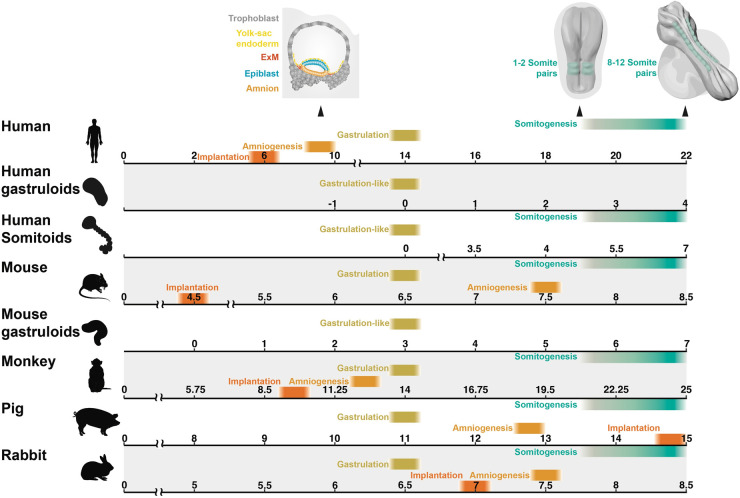
Comparing gastrulation across different species *in vivo* and *in vitro*. There are clear differences in the absolute and relative timing of differentiation events prior to and throughout mammalian gastrulation. Timelines indicate reported timings of amniogenesis, implantation, gastrulation commencement (or gastrulation-like commencement as is the case in *in vitro* models) as well as the timing of the first somite pair to the formation of the 10th somite pair summarised from multiple publications [5,8,17,20,21,24,25,44,46–48,73–75,80–83].

**Figure 2.**
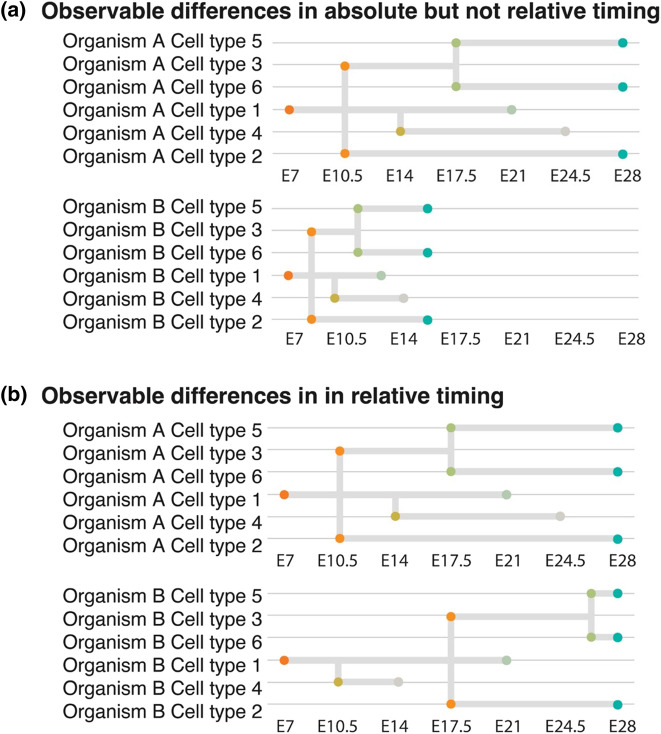
Relative and absolute differences in developmental tempo. Embryos may undergo the same series of differentiation events in the same order but within a different timeframe as is the case between organism A and B shown in (**a**). This constitutes a difference in the absolute but not relative differentiation timings. Observable differences in the order of differentiation events but not in the absolute time can also occur as demonstrated in (**b**). Dots indicate the point at which cell states diverge from a progenitor and theoretical ‘end-point' cell states. Timepoints on *y*-axis represent hypothetical days post-fertilisation.

Many characteristics of mouse gastruloids closely mirror that of 2D micropatterned human ESCs (hESCs) following the addition of exogenous WNT3A, which like mouse Wnt3, appears to be downstream of BMP4 [11–16]. Common characteristics include the specific spatial segregation of *SOX2*, *TBXT*, and *SOX17* demarcating ectodermal, mesodermal and endodermal domains, respectively, as well as localised induction of organiser gene expression such as *GSC*. These findings suggest a conserved mechanism of ‘WNT priming’ is sufficient to drive ‘primed' cells to self-organise and undergo differentiation processes reminiscent of gastrulation in both mice and humans. 3D human and mouse gastruloids [17] share many notable characteristics such as multi-axial organisation, anterior-posterior (A-P) elongation and cellular derivatives of all three germ layers. Despite the differences in the developmental timings of mouse and human embryos, as shown in Figure 1, gastruloids showed similar timing and spatial localisation of early lineage specification and HOX genes. Notably, however, both mice and human gastruloids did not produce cell types associated with anterior neural fates. Curiously, 72 h cultured human gastruloids transcriptionally align with 120 h mouse gastruloids which themselves are approximately equivalent to E8.25-stage mouse embryos [5,18–21]. Given that the starting state, media and timing of Chi induction differ, it is unclear whether the timings of human gastruloids are equivalent to mice. Furthermore, these comparisons were largely made using bulk-RNA seq and thus it is not possible to discern individual cell differentiation dynamics. However, these data suggest that mouse gastruloids transit from a naïve pluripotent state; equivalent to an E4.5 embryo [20,22] toward a state comparable to an E8.25 embryo (a 90 h difference) in 120 h, suggesting that mouse gastruloids take ∼30 h longer to reach the corresponding *in vivo* developmental stage. Human gastruloid development shows an even greater disparity compared with *in vivo* embryos as hESCs transition from a primed pluripotent state equivalent to E9–11 embryos [20,23] to a state that resembles an E17–19 embryo [17] in 72 h, ∼72 to 168 h faster than they would *in vivo*. This suggests a clear discrepancy between *in vitro* timings and *in vivo* timings due purely to extrinsic factors rather than the intrinsic properties of the cell. This also suggests that there is a clear distinction between cellular potential and fate; put simply what a cell can do may differ from what it does do. While transcriptionally gastruloids share many known features of gastrulation with their *in vivo* counterparts, it is important to note that as well as lacking extra-embryonic and anterior tissues, gastruloids do not correctly recapitulate many structures that characterise gastrulation, including a posterior ingressing primitive streak, a ventral endodermal layer, notochord, segmented somites and a neural tube. Gastruloids do, however, represent an attunable model system to study mammalian gastrulation events. Importantly, *in vitro* systems like gastruloids can facilitate comparisons of cells from different species outside of the confines of the embryo and therefore allow the untangling of extrinsic factors such as cell signalling from intrinsic cell properties (Figure 2).

## Cell intrinsic biochemical dynamics may underly developmental asynchronism

While earlier gastruloid models showed relatively simplistic tissue morphology, gastruloids cultured in extracellular matrix (ECM) support develop complex segmented somite structures [24] and closely recapitulate rostral-caudal patterning *in vivo*. Indeed, single-cell and spatial profiling demonstrate the formation of nascent and mature somitic cell types. Gastruloids can form a wide diversity of lineages including cardiac mesoderm, neuro-mesodermal progenitors (NMPs), mesenchyme, endothelium and allantois. The addition of bFGF, SB431542, with or without DMH1, limits the cellular diversity of gastruloids largely to somitic cell types [25,26]. Addition of BMP inhibitor LDN combined with Chi at pre-treatment can also limit gastruloid development largely to somitic cell types when provided with ECM support [27]. Curiously, the ECM support has little effect on the transcriptional profiles of these ‘somitoids/axioloids' yet develop organised somite segments in a pairwise fashion anterior to posterior in a manner which appears to recapitulate somite formation *in vivo* [26].

**Figure 3.**
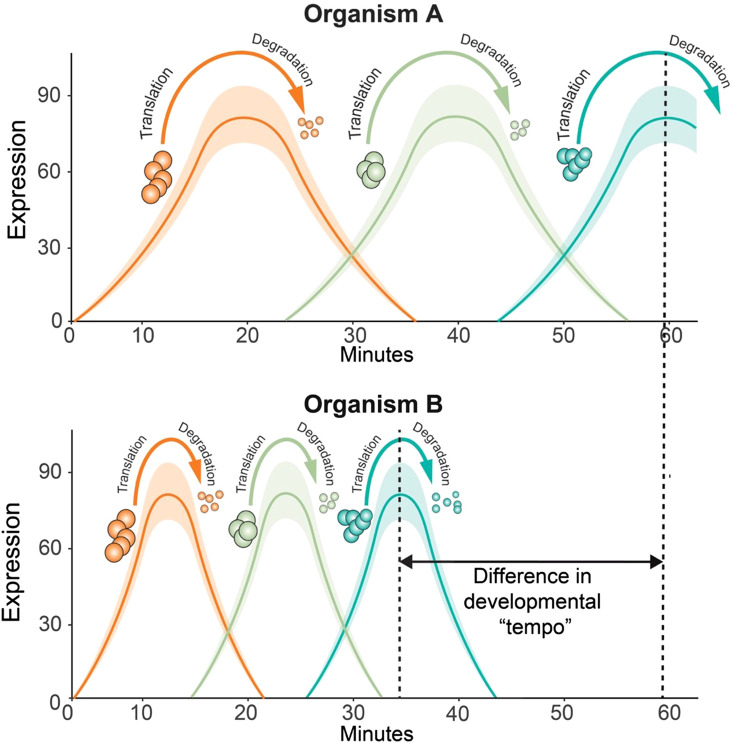
Protein half-lives may underly differences in developmental tempos. A schematic hypothetical of the translation of and degradation of three hypothetical proteins. In the two hypothetical organisms, the expression of each protein is dependent on the expression and degradation of the previous protein in order to progress from cell type 1 to cell type 2. In organism A it takes ∼60 min for protein 3 to reach peak expression while in organism B the increased rate of protein translation and degradation results in the process taking ∼25 min less in organism B. Only protein turnover is shown here however similar trends have been demonstrated between species regarding other biochemical processes for example RNA transcription whereby transcription, intron splicing and transcript degradation times may be shorter in organism A than organism B.

Utilising an *in vitro* model of somite development [28,29], Matsuda and colleagues [30] investigated developmental allochrony during somitogenesis. Employing a reporter for the somitogenesis master regulator *HES7*, they determined *in vitro* mouse pre-somitic mesoderm (PSM) oscillations were ∼2 h compared with human PSM oscillations which lasted in excess of 3 h, periods nearly identical with reports of their oscillation periods *in vivo* [31,32]. Further investigations suggested that the oscillation asynchrony was not due to sequence discrepancies between the mouse and human *HES7* orthologues but rather cell-autonomous differences in protein biochemistry, specifically the HES7 protein half-life was considerably shorter in mice [30]. This difference in protein turnover was also observed for other regulators of somitogenesis including GBX2, MSGN1, and TBX6 proteins. In contrast, CDX2, EVX1, and TBXT/Bra did not show any difference. In addition to protein half-life, transcription and intron delays were also observed in human HES7, TBX6, GBX2, and MSGN1 but not EVX1. These results suggest that the oscillation periods in human and mouse PSM are dependent on the biochemical properties including the timing of transcription, intron splicing and protein turnover of several key genes (an example of this scenario is shown in Figure 3). Consistent with the idea that intrinsic biochemical dynamics may underlie differences in the timing of cell differentiation, directed differentiation of mouse ESC toward motor neurons occurs at twice the speed of human ESC. As with Matsuda et al., neither signalling, genomic sequence of genes nor their regulatory elements correlated with the rate of differentiation. However, both protein stability and cell-cycle duration in human cells was around twice that of mouse [33].

A recent study [34] comparing somite oscillation timings in PSM derived from ESCs and induced pluripotent stem cells (iPSCs) from a diverse array of mammalian species including rabbit, cattle, rhinoceros, marmoset (ESCs) mouse, and human (iPSCs) [30]. Notably, these species represent animals with a wide range of adult body weights and gestation lengths [34] from three distinct phylogenetic clades: primates, glires, and ungulates. The study found that the segmentation clock oscillatory period did not scale with adult body weight but with embryogenesis length. As with humans and mice, oscillation length was largely correlated with both the splicing delay of the HES7 transcript and half-life of the protein, further supporting the notion that cell-autonomous differences in biochemistry may underly temporal discrepancies during cell differentiation across species.

Diaz-Cuadros et al. [35] demonstrated that mitochondrial activity through NAD+/NADH redox balance regulates segmentation clock timings in mouse and human cells acting upstream of protein synthesis rates. Manipulating metabolic rates by NADH oxidase overexpression or electron transport chain inhibition and altering the cellular NAD+/NADH ratio were able to increase or decrease oscillatory periods and global protein synthesis, respectively. Similarly, Iwata et al. [36] reported that species-specific timings in mouse and human cortical neuron maturation were linked to mitochondrial development and metabolic activity. Metabolic stimulation through lactate dehydrogenase inhibitors or free fatty acid addition was able to accelerate human neuronal differentiation. Conversely, inhibition of mitochondrial metabolism in mouse neurons led to decreased rates of differentiation. These studies suggest that metabolic activity may underly the previously described differences in cell biology that limit somitic and neuronal differentiation rates.

The development of so-called stem cell-derived embryos offers further insights into the processes of interspecies developmental control. Unlike gastruloids/axioloids/somitoids these aggregates are initially made up of embryonic and extra-embryonic cells [37–39]. Initially, created by mixing mouse trophoblast stem cells (TSC), extra-embryonic endoderm (XEN) cells and mESC [37], later methods used naïve cells transiently expressing either *Cdx2* or *Gata4* in place of TSC and XEN cells, respectively [40]. These aggregates, like gastruloids, are capable of self-organising however, they form far more complex structures resembling embryos. In the case of mice, stem-cell-derived embryos have a conical shape with the extra-embryonic ectoderm atop a conical epiblast-like structure which is surrounded by a visceral endoderm-like structure [37]. Human stem cell-derived embryos which correspondingly form structures which are more akin to that of a developing human, cavitate and form amnion-like structures atop the epiblast, a hypoblast-like layer beneath the epiblast and extensive extra-embryonic mesoderm (ExM) [38,39]. Mouse stem cell-derived embryos, if cultured in ex utero media, are able to develop past primary gastrulation and form remarkably complex tissues including headfolds with defined forebrain and midbrain, a beating heart, a neural tube, somites, a tail bud, a gut tube, and primordial germ cell-like cells (PGC-LCs) [41,42]. Curiously, these structures form in the absence of exogenous ECM support suggesting that extra-embryonic tissues may be linked to embryo morphology. While to date there have been no direct comparisons of mouse and human stem cell-derived embryos these present an interesting model to understand the interplay between embryonic and extra-embryonic tissues during gastrulation in different species.

## Single-cell sequencing provides insights into developmental control across species

Recent single-cell atlases, detailing gastrulation stage embryos in mice, humans, monkeys, pigs, and rabbits [21,43–48]offer unparalleled views of early differentiation dynamics in diverse species. By facilitating direct, quantitative comparison of transcriptional landscapes, these atlases can be used to unravel conserved and divergent aspects of developmental control. These comprehensive atlases in combination with functional work present a powerful tool for investigating the molecular underpinnings of embryonic patterning, differentiation, and morphogenesis, as well as their perturbations in developmental disorders.

In 2019, the first detailed map of mouse gastrulation and early organogenesis was produced, charting cellular differentiation from pluripotency to major embryonic lineages [21]. This study set the groundwork for understanding cellular differentiation in mice. Soon after, the sequencing of a mid-gastrulation human embryo [43] allowed for the first direct comparison of germ-layer progenitors between these species. The authors collected single cells from mouse embryos at time points ranging from 6.5 to 8.5 days post-fertilization and constructed a molecular map of cellular differentiation providing a critical reference for cellular differentiation in mice. Comparing the transition from epiblast to nascent mesoderm in the human gastrula with analogous populations in mice [21] highlighted both common and divergent features during this transition. For example, both species display down-regulation of *CDH1* and concomitant up-regulation of *TBXT* and *SNAI1* during epithelial-to-mesenchymal (EMT). Differences included human-specific expression of mesenchymal marker *SNAI2* and divergent expression patterns of *FGF8* and *FGF2*. *In vitro* experiments confirmed these observations, as human cells showed a requirement for MEK signalling for the down-regulation of *FGF2* and subsequently, EMT [43].

Another interesting observation was the unexpected presence of advanced blood progenitors in the CS7 human embryo, not present in mice until around E8.5, which morphologically resembles late CS10. Despite the maturity of blood progenitors, the epiblast and primitive streak cell clusters had a greater semblance to their E7 and E7.5 mouse counterparts, respectively. Given that the human dataset represents a single embryo and less than 1200 cells this prevents conclusive interpretations of these observations. However, these findings, if reproducible, are a salient example of how even closely related species such as humans and *macaques* can differ in their transcriptional programs. Programs such as haematopoiesis, which appear to occur at an accelerated rate in humans relative to mice, suggest that relative cell differentiation tempos may not always scale with embryogenesis length.

More recently, a single-cell transcriptome atlas of *Cynomolgus* monkey gastrulation and early organogenesis [44] from CS8 to CS11 provided the first comparative analyses between primate and rodent embryos [21] as well as 2D human gastruloid models [49,50] and somitoids [25]. The authors show that primate cells transitioning toward NMP/PSM have higher expression of Hippo signalling genes. Validation experiments using monkey, mouse, and human *in vitro* PSM differentiation models revealed that primate cells had higher expression levels of *MLLT3* and *FOSB*. Furthermore, increased levels of nuclear *YAP1* in primate PSM-like cells suggest that primate cells have lower Hippo kinase activities. Furthermore, inhibition of the Hippo pathway by LPA severely impaired mouse but not monkey or human PSM differentiation. Curiously, when comparing their *in vivo* data to different *in vitro* models the authors noted that the up-regulation of the Hippo pathway-associated genes was not recapitulated in Human somitoid models [25]. This is a particularly interesting observation given the known role of Hippo signalling in regulating developmental patterning through morphological-mechanical cues [51–53]. These observations highlight the continued need for *in vivo* references to validate and contextualise *in vitro* findings.

## New models of mammalian development provide unique perspectives on interspecies developmental control

Mayshar and colleagues created a time-resolved flow model of gastrulation using rabbit and mouse embryos [45,46]. The authors identified a highly conserved ‘regulatory core' of 75 transcription factors that regulate cell states. Furthermore, there was a very close alignment of stable transcriptional states suggesting that despite the differences in embryo morphology and implantation, cells follow a similar differentiation trajectory. Despite the apparently conserved BMP4-WNT hierarchy seen in *in vitro* systems [11–16], the group found no evidence of trophectoderm produced BMP4. They did, however, find evidence of hypoblast-derived BMP2 which along with polarised expression of the BMP and WNT inhibitor CER1 was suggested to be the main driver of A-P axis formation in rabbits. This finding is also consistent with data in pigs [48,54], and while it is unclear to what extent BMP2 and 4 are interchangeable, it does suggest that the extra-embryonic ectoplacental cone has evolved unique signalling properties in mice.

Comparisons of the transcriptional states showed that despite very similar developmental times, there were notable periods of transcriptional asynchrony between mice and rabbits. For example, the transcriptional profiles of rabbit embryos between E6 to E7.25 had a stronger correlation with E6–E6.75 mouse embryos suggesting that the embryos took longer to reach similar transcriptional states. However, by E7.5 both rabbit and mouse embryos were at a similar transcriptional state. In other words, despite a head start in differentiation, the rabbit cells caught up. This ‘hourglass’-like bottleneck is exemplified by cells of the anterior primitive streak (APS) which appear to accumulate more rapidly during a comparable developmental period. Furthermore, these endoderm-fated cells and their mesoderm-fated counterparts in the primitive streak appear to rapidly acquire a mutually exclusive expression of fate determinants such as *FOXA2* and *MSX1* than mice.

The pig gastrulation and early organogenesis dataset [48] uncovered several findings relevant to the broader discourse on interspecies developmental control, as for the first time we were able to make quantitative comparisons of gastrulation between representatives of rodents, artiodactyls, and primates. We identified a considerable overlap within cell-type-specific transcriptional programs across homologous cell types across pig, monkey, and mouse cells, with surprisingly few cell-type-specific genes demonstrating opposing expression profiles. We did, however, note that numerous genes exhibited differential expression between a given cell type versus its homologue in another species when the analysis was not restricted to cell-type-specific genes. Many of the identified genes were part of pathways related to cell behaviours including growth, proliferation, differentiation, and morphogenesis such as the MAPK, PI3K/Akt signalling and focal adhesion pathways. While these may contribute to known differences in size, cell-cycle length and morphology, this also may suggest greater evolutionary constraints on factors regulating the formation of specific cell types.

We also identified differences between cell differentiation dynamics between pigs, mice, and monkeys using the single-timepoint human embryo [43] as a reference. While the human embryo morphologically resembles a mid-gastrula embryo, the ExM closely aligned with E15 (10 Somite stage) ExM in pigs. This suggests that ExM is not only more extensive in human embryos but also undergoes accelerated maturation compared with other cell types such as epiblast or primitive streak cells. Similar observations were made comparing human ExM cells to their mouse counterparts. In contrast, the majority of human cell types matched a single stage in primates. A similar trend was found with yolk-sac endoderm which also appeared to mature faster in primates than mice and pigs. Given the role of extra-embryonic tissues in implantation, it stands to reason that such enhanced maturation was necessary to facilitate the comparatively early implantation of primates. This suggests that in primates the timing of maturation of these extra-embryonic tissues has diverged from the trunk of mammals. Interestingly, both tissues that appear to mature faster in humans, the ExM and yolk-sac endoderm, are involved in the formation of the blood island and patterning of early blood progenitors [55–58]. The presence of advanced blood progenitors in the CS7 human embryo was also noted by Tyser et al.[43], it may be that the enhanced maturation of these extra-embryonic tissues could not occur without the ‘knock-on' effect of earlier blood maturation.

Our results [48] suggested that cell behaviour and dynamics differed across species but that mechanisms of differentiation are likely conserved. Following this logic, we investigated whether large mammals formed mesodermal and endodermal progenitors via discrete mechanisms as has been demonstrated in mice [45,59,60]. Indeed, this has been an area of controversy within mammalian embryology as the idea that mesodermal and endodermal germ layers originate from a common mesendodermal progenitor, a hypothesis seemingly validated *in vitro* in hESCs [61–64]. Intriguingly, we [48] found corroborative evidence for the rodent model in pigs, and further support for its relevance to human cells via a series of *in vitro* experiments, resolving prior inconsistencies. This further supports the notion that intrinsic programs are highly conserved while extrinsic signals may alter differentiation dynamics.

## Intrinsic and extrinsic factors underlie differences in developmental control

There have been several hypotheses that have attempted to explain interspecies differences in developmental control. One hypothesis suggested that differentiation may be reliant on a set number of cell divisions, and known differences in cell-cycle length may account for temporal discrepancies [65]. However, it has been demonstrated that differentiation can occur in the absence of cell division [66,67]. Hypotheses which suggest species-specific differences in intrinsic cell biochemistry including transcription, splicing and translation rates may indeed be the rate-limiting factor in the formation of cell types such as somites [30,34] and specific types of neurons have a wealth of evidence to support them [33]. More recently it has been posited that metabolic rates, may determine the timing of these biochemical reactions [35,36]. While the evidence presented may suggest that metabolic rates may present a rate limitation, it is also true that metabolism itself is dependent on many extrinsic factors [36,68]. For example, a study [69] utilising fibroblasts collected from 10 different mammalian species showed that under identical culture conditions cells exhibited similar metabolic rates, and these rates are dependent on oxygen concentrations. Indeed, Iwata and colleagues were also able to increase the rate of neuronal differentiation by modulating fatty acid availability [36,69,70]. Recent studies creating human-pig chimaeras have demonstrated the ability of genetically modified human cells to differentiate at rates comparable to their pig hosts [71,72]. Maeng et al. [71] demonstrated that TP53 KO human iPSCs form mature skeletal muscle in all 27 somites of MYF5/MYOD/MYF6 KO E20 pig embryos. This is particularly striking as human cells may have only begun to form their first somite within the human embryo [73–75]. Similarly, the work by Wang et al. demonstrates *MYCN* and *BCL2* overexpressing human iPSCs can form mesonephros in *SIX1/SALL1* null E25 pig embryos which are roughly equivalent comparable to an E40 human embryo [72–75]. Given that the genetic modifications in both studies enhance cell competition through repression of apoptosis rather than through enhancements of cell biochemistry [76], this suggests that extrinsic signalling environments can affect the rate at which cells differentiate. Another recent study [77] demonstrated that *in vitro*-produced human neural rosettes could form interneurons from a primed pluripotent state in 13 days, a process which would take ∼20–27 days in the human foetus [72–75,77]. Curiously, human rosettes grafted into faster-developing chick hosts failed to differentiate at an increased rate. It was however found that the human grafts slowed the differentiation of some of the surrounding chicken neurons. Furthermore, iso-chronically isolated neural cells grafted into chicks displayed even slower differentiation than their *in vitro* counterparts [77]. Lastly, a study [78] utilising chimeric culture experiments, demonstrated that co-culture of human and mouse pluripotent cells during neuronal differentiation accelerated the maturation of human neurons. It is therefore clear that the speed of human differentiation *in vivo* does not represent a maximum differentiation rate and that there is an inherent plasticity to embryonic cells whereby they are able to adapt to a variety of signalling and metabolic environments, however, the limit to this plasticity is less clear. Given that *in vivo*, methods and timings of implantation, and extra-embryonic tissues vary significantly across mammals [4,6,21,43,45–48]. Furthermore, uterine oxygen levels and nutrient availability differences between species are unclear. Consequently, it is plausible that these diverse extrinsic factors represent an evolutionary tactic to expedite development. However, no studies to date have demonstrated that extrinsic factors can enhance human cell differentiation rates to match those observed in mice. Indeed, intrinsic factors may present a rate-limiting factor setting an ‘upper limit' on the pace of gastrulation.

## Future directions

While the studies outlined in this review have shed light on aspects of developmental control across mammalian species, several key directions beckon for future study. First, in order to validate existing observations, the requirement for more datasets becomes essential. The discrepancies observed across species have so far relied on relatively low sample numbers. Given the limited number of samples *in vitro* and *in vivo*, the presence of batch effects is a particular concern and can potentially confound and mislead interpretations. At present there is a risk that in attempting to rectify these batch effects, we may inadvertently correct genuine interspecies differences. Enhanced, more consistent datasets could obviate this problem, allowing us to disentangle genuine biological differences from technical artefacts. In addition to increasing the number of datasets of characterised species, unravelling the evolution of specific mechanisms of developmental control requires expanding our catalogue of studied species. This expansion will help elucidate the interplay between intrinsic cellular biochemical dynamics and broader interspecies differences, presenting a clearer picture of the processes governing gastrulation.

Beyond single-cell transcriptomics, spatial transcriptomics combined with up-to-date modelling techniques will be instrumental in elucidating the influence of morphological differences on differentiation events. Lastly, comparative *in vitro* studies in tandem with *in vivo* studies are central to understanding extrinsic cues vs. intrinsic differentiation capacities of cells. Indeed, the availability of ESC cell lines from multiple mammals offer excellent models for cross-species comparisons [79]. However, *in vitro* comparisons, in particular, may be hampered by variabilities in culture conditions. A concerted effort to standardise culture conditions, such as defining identical media for each species, could ensure that observed differences stem from inherent cellular properties rather than extrinsic culture-induced effects.

## Summary

- Gastrulation represents one of the most important periods of development. A fuller understanding of gastrulation can help us understand congenital defects in humans as well as species evolution.
- Single-cell atlases of gastrulation demonstrate both absolute and relative differences in differentiation dynamics between species.
- *In vitro* and *in vivo* evidence from multiple mammalian species suggest intrinsic differences in cell biochemistry and extrinsic cell–cell signalling influence differentiation dynamics.
- Additional single-cell and spatial datasets, as well as *in vitro* experiments in a range of species should be the focus of future studies to decipher the intricate balance between intrinsic cellular programs and extrinsic signals in interspecies developmental control.

## Open Access

Open access for this article was enabled by the participation of University of Nottingham in an all-inclusive *Read & Publish* agreement with Portland Press and the Biochemical Society under a transformative agreement with JISC.

## Abbreviations

EMT: epithelial-to-mesenchymal transition
ESC: embryonic stem cells
ExM: extra-embryonic mesoderm
hESC: human pluripotent stem cells
iPSCs: induced pluripotent stem cells
mESC: mouse embryonic stem cells
NMPs: neuro-mesodermal progenitors
PGC-LC: primordial germ cell-like cells
